# Langerhans-type and monocyte-derived human dendritic cells have different susceptibilities to mRNA electroporation with distinct effects on maturation and activation: implications for immunogenicity in dendritic cell-based immunotherapy

**DOI:** 10.1186/1479-5876-11-166

**Published:** 2013-07-09

**Authors:** David J Chung, Emanuela Romano, Katherine B Pronschinske, Justin A Shyer, Milena Mennecozzi, Erin T St Angelo, James W Young

**Affiliations:** 1 Laboratory of Cellular Immunobiology, New York, NY, USA; 2 Swim Across America Laboratory, New York, NY, USA; 3 Adult Bone Marrow Transplant Service, Division of Hematologic Oncology, New York, NY, USA; 4 Department of Medicine, New York, NY, USA; 5 Immunology Program, Sloan-Kettering Institute for Cancer Research, New York, NY, USA; 6 Memorial Sloan-Kettering Cancer Center, New York, NY, USA; 7 Weill Cornell Medical College, New York, NY, USA; 8 Centre Hospitalier Universitaire Vaudois, CHUV-BH06, Rue du Bugnon 46, 1011, Lausanne, Switzerland/Suisse; 9 European Commission, Joint Research Centre, Ispra (VA) 21027, Italy

**Keywords:** mRNA electroporation, Dendritic cells, Cancer, Immunotherapy

## Abstract

**Background:**

mRNA electroporation of dendritic cells (DCs) facilitates processing and presentation of multiple peptides derived from whole antigen, tailored to different HLA molecules. Clinical responses to electroporated moDC vaccines, however, have been suboptimal. Human Langerhans-type DCs (LCs) are the most potent conventional DC subtype for inducing CD8^+^ cytotoxic T lymphocytes (CTLs) *in vitro*. We recently demonstrated that Wilms’ tumor 1 (WT1) mRNA-electroporated LCs are superior to moDCs as stimulators of tumor antigen-specific CD8^+^ CTLs, even though they are comparable stimulators of allogeneic T cell proliferative responses. A detailed comparative evaluation of the effects of mRNA electroporation on LCs versus moDCs, however, is needed.

**Methods:**

Immature and partially-matured human moDCs and LCs electroporated with mRNA were compared for transfection efficiency, phenotypic changes, viability, retention of transgene expression after cryopreservation, and immunogenicity. Student t test was used for each pairwise comparison. One-way analysis of variance was used for multiple group comparisons.

**Results:**

Transfection efficiency after electroporation with enhanced green fluorescent protein (eGFP) mRNA was higher for immature than for partially-matured moDCs. In contrast, transfection efficiency was higher for partially-matured than for immature LCs, with the additional benefit that electroporation itself increased maturation and activation of CD83^+^HLA-DR^bright^ LCs but not moDCs. Electroporation did not impair final maturation and activation of either DC subtype, after which both mRNA-electroporated LCs and moDCs were functionally similar in stimulating allogeneic T cell proliferation, a standard assay of DC immunogenicity.

**Conclusions:**

These findings support mRNA electroporation of DCs, and in particular LCs, as an effective non-viral method to stimulate specific, potent CD8^+^ CTL responses. The differences between LCs and moDCs regarding this form of antigen-loading have important implications for DC-based immunotherapies.

## Introduction

Effective therapeutic cancer vaccination requires the optimization of tumor antigen presentation by antigen-presenting cells (APCs) to induce strong antigen-specific T cell responses, especially CD8^+^ cytolytic T lymphocytes (CTLs) with a sufficiently broad repertoire and immunologic memory [1]. Human conventional dendritic cells (DCs) are the most potent APCs and are critical to the onset of immunity. DCs prime T cell responses by coupling antigen to all the requisite co-stimulatory, cytokine, and chemokine signals required for the activation of naive and resting T cells [2]. Numerous studies have demonstrated the feasibility of DC-based immunization to induce host responses against tumors [3].

Electroporation of DCs with mRNA encoding specific tumor-associated antigens is an effective non-viral method to stimulate T cell responses *in vitro* and *in vivo*[4-11]. This method of antigen loading, which facilitates processing and presentation of multiple class I and II MHC-restricted epitopes from the translated protein [12], is more efficient than peptide pulsing and less problematic than retroviral transgenes, which carry the risk of genome integration [13]. mRNA electroporation also allows individuals of any HLA type to process and present peptides tailored to their own MHC molecules.

Monocyte-derived DCs (moDCs) are the most commonly used DC subtype in cancer vaccines, and the induction of tumor-specific CTLs *in vitro* by mRNA-transfected moDCs has been reported in several studies [4-10]. Clinical responses to moDC-based vaccines, however, have not always achieved optimal stimulation of antigen-specific CTLs; and data on clinical responses to vaccination with mRNA-electroporated moDCs are limited [7,11].

Human Langerhans-type DCs (LCs) derived from CD34^+^ hematopoietic progenitor cells are the most potent conventional DC subtype for stimulating CD8^+^ CTLs *in vitro*[14-17]. We recently showed that WT1 mRNA-electroporated LCs are superior to moDCs as stimulators of antigen-specific CTLs *in vitro,* using an IL15R-α/IL15/pSTAT5-dependent mechanism [16]. LCs synthesize abundant IL15 mRNA and protein, whereas moDCs are dependent on exogenous IL15 for stimulating comparably potent WT1-specific CTLs [16]. The effects of mRNA electroporation on moDCs have been described [18]. A detailed comparative evaluation of the effects of mRNA electroporation on LCs versus moDCs is still needed, however.

In this study, we compared moDCs and LCs after mRNA electroporation for transfection efficiency, phenotypic changes, viability, retention of transgene expression after cryopreservation, and allo-stimulatory capacity. Our findings clearly demonstrate that the maturation state of moDCs and LCs differentially affects their susceptibility to electroporation, and electroporation itself has a useful maturational effect on LCs but not moDCs. These findings underscore the importance of tailoring electroporation conditions to specific DC subtypes when designing DC-based immunotherapies.

## Methods

### Blood samples

Peripheral blood mononuclear cells (PBMC) or granulocyte colony stimulating factor (G-CSF)–elicited CD34^+^ hematopoietic progenitor cells (HPC) were obtained from healthy volunteers or allogeneic hematopoietic stem cell transplant donors. Buffy coats purchased from the Greater New York Blood Center, American Red Cross, were also used as a source of cells from healthy donors. Biospecimen sample collection and use adhered to protocols approved by the Institutional Review and Privacy Board of Memorial Hospital, Memorial Sloan-Kettering Cancer Center (MSKCC).

### Media, serum, and non-cytokine reagents

For moDC cultures, complete RPMI 1640 was supplemented with 10 mM HEPES (N-2-hydroxyethylpiperazine-N’-2-ethanesulfonic acid), 1% penicillin/streptomycin (Media Laboratory, MSKCC), 50 μM 2-mercaptoethanol (GibcoBRL Life Technologies), 2 mM L-glutamine (GibcoBRL), and heat-inactivated, autologous single-donor or pooled human serum (1% or 10% vol/vol). For LC cultures, X-VIVO 15 (BioWhittaker) was only supplemented with cytokines (see below). All media and reagents were endotoxin-free.

### Generation of moDCs and LCs with recombinant human cytokines

MoDCs were generated from PBMCs, and LCs were generated from G-CSF–elicited CD34^+^ HPCs. Media, media supplements, and cytokines were exactly as published [14]. In brief, for immature moDC generation, tissue culture plastic adherent CD14^+^ monocytes were cultured in complete RPMI-1% normal human serum (NHS) with GM-CSF and IL-4 for 5 to 6 days. For immature LC generation, CD34^+^ HPCs were cultured in serum-free X-VIVO 15, supplemented with GM-CSF, TGF-β, and TNF-α, to which c-*kit*-ligand and FLT-3-ligand were added for only the first 5 to 6 days of a 10- to 12-day culture. Terminal maturation of moDCs and LCs was induced with a combination of TNF-α, IL-1β, IL-6, and prostaglandin E_2_.

### T lymphocytes

T cells were obtained from tissue culture plastic-nonadherent PBMCs, further purified by nonadherence and elution from nylon wool columns (Polysciences). This achieved >95% purity without bystander activation of T cells.

### Plasmids

An eGFP-containing plasmid, pGEM4Z/eGFP/A64, was obtained from Dr. E. Gilboa, University of Miami. For WT1, an EcoRI insert encoding WT1 cDNA, derived from the pUC119 plasmid (Riken Bioresource), was cloned into a pGEM-4Z vector (Promega). Plasmids were propagated in Max Efficiency DH5-α competent cells (Invitrogen) and purified using a Plasmid Maxi Kit (QIAGEN).

### Production of in vitro–transcribed mRNA

For eGFP mRNA transcription, the pGEM4Z/eGFP/A64 plasmid was linearized with SpeI (New England Biolabs) before mRNA transcription *in vitro*, which was performed with T7 RNA polymerase (mMessage mMachine T7 kit; Ambion). For WT1 mRNA transcription, the pGEM-4Z/WT1 plasmid was linearized with HindIII (New England Biolabs) before mRNA transcription *in vitro*, which was performed with SP6 RNA polymerase (mMessage mMachine SP6 kit; Ambion). For both eGFP and WT1 mRNA, agarose gel electrophoresis confirmed production of full-length capped mRNA, and spectrophotometry measured mRNA concentration.

### Electroporation of cells

Immature moDCs were electroporated on day 5–6 and immature LCs on day 10–11. Partially-matured moDCs were electroporated on day 7–8 and partially-matured LCs on day 12–13, reflecting the different times required to generate these two DC subtypes *in vitro*[14,15]. After harvesting, cells were washed twice and resuspended in OptiMEM (Gibco, Invitrogen) at 15×10^6^ cells/ml. 200 μL of cell suspension were then mixed with 20–40 μg mRNA and electroporated in a 2 mm gap cuvette at 650–900 V for 1–3 pulses at 0.5 msec/pulse, using a BTX ECM 830 square-wave electroporator (BTX Harvard). After electroporation, cells were immediately transferred to culture to minimize cell death. While in culture, immature cells were partially or terminally matured by exposure to inflammatory cytokines for 24 or 48 hours, respectively.

### Phenotypic and eGFP expression analyses by flow cytometry

Fluorescein (FITC)-, phycoerythrin (PE)-, PE-cyanine-7–, and allophycocyanin (APC)–conjugated mouse anti–human monoclonal antibodies included anti-CD80, anti-CD83, anti-CD86, and anti–human histocompatibility leukocyte antigen (HLA)–DR (BD Biosciences). Nonreactive isotype-matched antibodies (Becton Dickinson) were used as controls. Post-electroporation eGFP expression was assessed, compared with expression by mock-electroporated DCs. Flow cytometry analyses used a Cytomics FC500 flow cytometer (Beckman Coulter) or LSRFortessa cell analyzer (BD Biosciences). Gates were set for collection and analysis of at least 20,000 live events. Data were analyzed with FlowJo software (TreeStar).

### Allogeneic mixed leukocyte reactions (MLRs)

Mock-, eGFP mRNA-, and human WT1 mRNA-electroporated moDCs or LCs were separately added in serial doses (1:10 to 1:1000, moDC:T) to triplicate wells of 1 × 10^5^ allogeneic T cells in 96 round-bottomed well plates (Corning Life Sciences). Final volume was 100 μL/well of complete RPMI-10% heat-inactivated, NHS serum. Responder allogeneic T cell proliferation was measured by either: 1) incorporation of methyl-[3H]thymidine ([3H]TdR, 1 μCi/well; New England Nuclear, Division of PerkinElmer Life Sciences) during the last 8 hours of a 5-day culture, as measured in a beta scintillation counter (Betaplate; Wallac, Division of PerkinElmer Life Sciences), or 2) colorimetric assay according to manufacturer's instructions (CellTiter96 Aqueous One Solution Cell Proliferation Assay MTS; Promega).

### Statistics

Student t test was used for each pairwise comparison. One-way analysis of variance was used for multiple group comparisons. A *P* value less than .05 was considered statistically significant.

## Results

### The transfection efficiency of mRNA electroporation varies with the maturation status of moDCs and LCs

**Figure 1:**
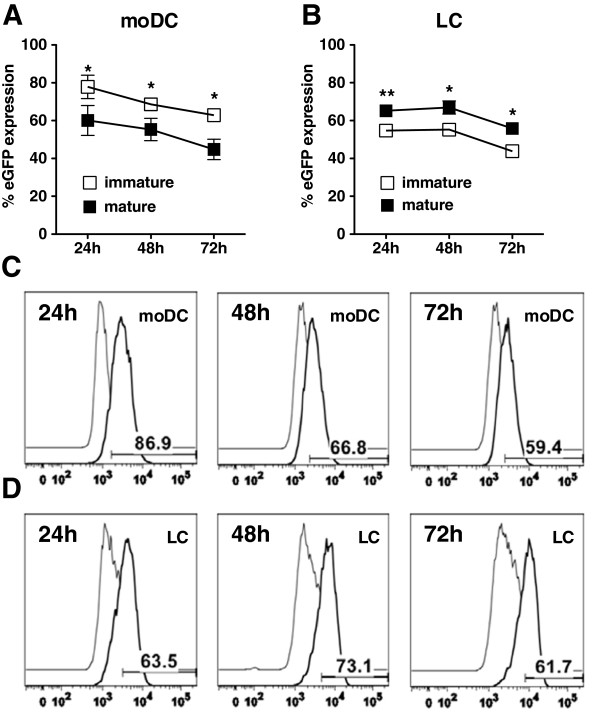
**The maturation status of moDCs and LCs affects mRNA-electroporation transfection efficiency.** Immature (□) and partially-matured (▤) moDCs **(A, C)** and LCs **(B, D)** were electroporated with eGFP-encoding mRNA. The transfection efficiency for each experimental group was assessed by flow cytometry at the indicated time points. Pooled data (mean ± SD, n = 6 independent experiments) are shown for moDCs **(A)** and LCs **(B)**. *P < .05 and **P < .01 for comparisons between immature and mature groups. Representative histograms of eGFP mRNA-electroporated immature moDCs **(C)** and partially-matured LCs **(D)** from one of six independent experiments are shown.

Immature and 24-hour, partially-matured moDCs and LCs were electroporated with eGFP mRNA. After electroporation, cells were immediately returned to culture for at least 24 hours of maturation before being assessed for eGFP expression as an index of transfection efficiency. As shown in Figure 1A, transfection efficiency was higher for immature than for partially-matured moDCs (peak value at 24 hours: 77.9 ± 12.4% for immature cells and 59.4 ± 15.4% for partially-matured cells). In contrast, transfection efficiency was higher for partially-matured than for immature LCs (Figure 1B; peak value at 48 hours: 67 ± 6.9% for partially-matured cells and 55.2 ± 2.9% for immature cells). Thus, both the type and maturation status of DCs influence mRNA transfection efficiency.

**Figure 2:**
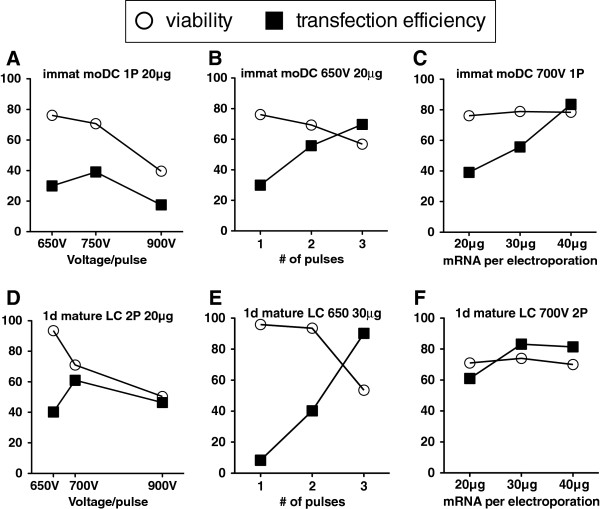
**Optimization of mRNA-electroporation conditions.** Immature moDCs **(A-C)** and partially-matured LCs **(D-F)** were electroporated with eGFP-encoding mRNA under different conditions of set voltage, number of electroporation pulses, or amount of mRNA. Only one of the three parameters was varied in any given set of tested conditions, as summarized in each panel. Cells were assessed for viability (○) by trypan blue exclusion and transfection efficiency (▤) by flow cytometry.

Optimal electroporation parameters for immature moDCs and partially-matured LCs were determined by varying set voltage, number of electroporation pulses, and amount of mRNA to maximize transfection efficiency while minimizing cell loss. For immature moDCs, best results were achieved with 700 V, 1 pulse, and 40 μg mRNA. For partially-matured LCs, best results were achieved with 700 V, 2 pulses, and 30 μg mRNA. Results are summarized in Figure 2.

### Immature LCs, in contrast to immature moDCs, display a more mature phenotype after mRNA electroporation

**Figure 3:**
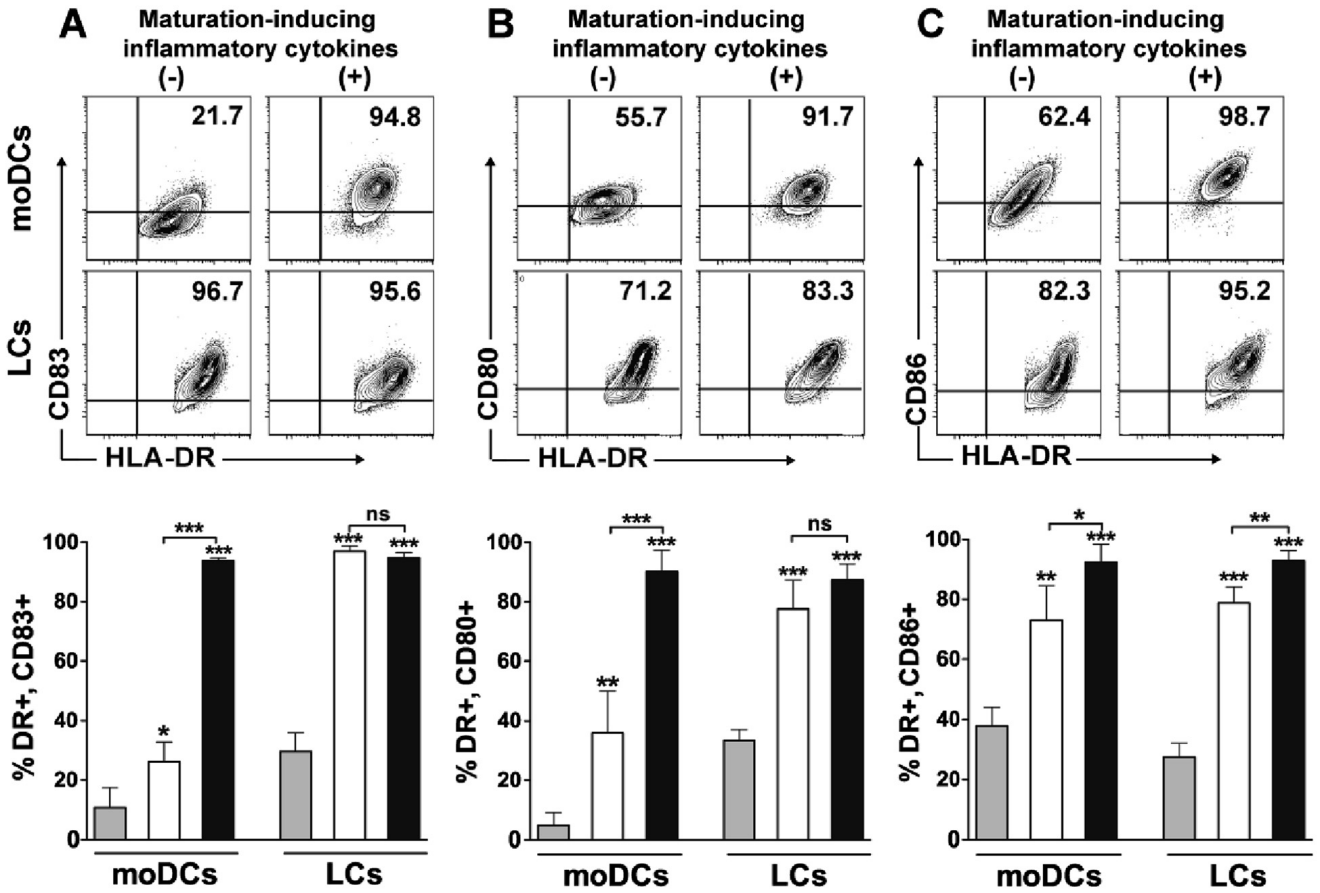
**mRNA electroporation induces the maturation and activation of LCs to a greater magnitude than for moDCs.** Immature moDCs and LCs were electroporated with eGFP mRNA and then cultured with (+) or without (−) standard maturation-inducing inflammatory cytokines. After 24 hours, cells in each experimental group were compared with pre-electroporation controls by flow cytometry for the expression of phenotypic markers of DC maturation and activation, based on the upregulation of **(A)** CD83, **(B)** CD80, and **(C)** CD86, respectively. Representative dot plots of eGFP mRNA-electroporated moDCs and LCs from one of three independent experiments are shown in the top row. Pooled data for each experimental group (mean ± SD, n = 3 independent experiments) are shown in the bottom row (gray bar = pre-electroporation control, white bar = post-electroporation without inflammatory cytokines, black bar = post-electroporation with inflammatory cytokines). *P < .05, **P < .01, ***P < .001, relative to pre-electroporation control, except where brackets indicate comparisons between post-electroporation groups.

Immature moDC and LCs electroporated with eGFP mRNA were incubated with or without standard inflammatory cytokines. After 24 hours, DCs were assessed by flow cytometry for the upregulation of the maturation and activation markers, CD83, CD80, and CD86 [14,15]. Electroporation had a mild direct effect on moDC maturation based on expression of the prototypical DC maturation marker, CD83 (Figure 3A; 10.68 ± 3.37% pre-electroporation and 26.13 ± 3.34% post-electroporation), whereas electroporation markedly increased the maturation of CD83^+^HLA-DR^bright^ LCs (Figure 3A; 29.62 ± 2.8% pre-electroporation and 96.92 ± 0.81% post-electroporation). Electroporation induced greater overall CD80 expression by LCs (Figure 3B; 33.36 ± 1.65% pre-electroporation and 77.03 ± 5.54% post-electroporation) than moDCs (Figure 3B; 4.9 ± 2.1% pre-electroporation and 35.88 ± 7.11% post-electroporation); fold increase of CD80 expression was greater for moDCs (7.3-fold increase for moDC and 2.3-fold increase for LCs). Electroporation had a similar effect on CD86 expression by moDCs (Figure 3C; 37.83 ± 3.12% pre-electroporation and 73 ± 5.77% post-electroporation) and LCs (Figure 3C; 27.44 ± 2.11% pre-electroporation and 78.65 ± 3.12% post-electroporation). For either DC subtype, electroporation did not impair terminal, inflammatory cytokine-induced maturation.

### Cell loss and viability of moDCs and LCs after electroporation and cryopreservation

**Figure 4:**
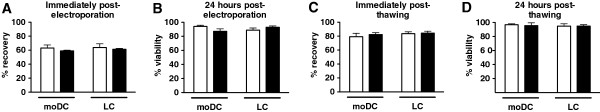
**Cell recovery and viability of moDCs and LCs after electroporation and cryopreservation.** Immature (□) and partially-matured (▤) moDCs and LCs were electroporated with eGFP-encoding mRNA, without purification by HLA-DR selection a priori. **(A)** Immediate post-electroporation viable cell recovery relative to the total number of cells electroporated was assessed by trypan blue exclusion (mean ± SD, n = 4 independent experiments). **(B)** Surviving cells from **(A)** were returned to culture, and cell viability was assessed 24 hours after electroporation, relative to initial number of cells returned to culture after electroporation (mean ± SD, n = 4 independent experiments). **(C)** After at least 24 hours in culture, cells from **(B)** were cryopreserved. Upon subsequent thawing, immediate post-thaw viability was assessed relative to initial number of cells frozen (mean ± SD, n = 4 independent experiments). **(D)** After 24 hours in culture, thawed cells from **(C)** were reassessed for viability (mean ± SD, n = 4 independent experiments).

Electroporation of cells can induce cell death due to the deleterious effects of direct electrical current and disruption of the cell membrane. Electroporation of immature and partially-matured moDCs and LCs resulted in up to approximately 40% initial cell death, as determined by trypan blue exclusion on direct hemacytometer counts (Figure 4A).

After electroporation, DCs were returned to culture for 24 hours, and cell viability was reassessed. Immature and mature moDCs and LCs demonstrated >85% viability (Figure 4B; 94.2 ± 3.0% for immature moDCs and 88.7 ± 6.3% for immature LCs; 87.1 ± 7.1% for partially-matured moDCs and 92.9 ± 3.8% for partially-matured LCs).

The viability of both DC subtypes was further evaluated after cryopreservation, with no significant difference in survival between moDCs and LCs immediately after thawing (Figure 4C; 79.2 ± 9.8% for immature moDCs and 83.6 ± 5.4% for immature LCs; 82.3 ± 5.2% for partially-matured moDCs and 84.4 ± 5.4% for partially-matured LCs) or after thawing and resting for 24 hours (Figure 4D; 96.8 ± 2.7% for immature moDCs and 94.8 ± 6.7% for immature LCs; 95.5 ± 8.4% for partially-matured moDCs and 94.9 ± 4.2% for partially-matured LCs). These anticipated yields are reproducible and inform the required number of starting cells.

### MoDCs and LCs electroporated with mRNA retain transgene expression after cryopreservation

**Figure 5:**
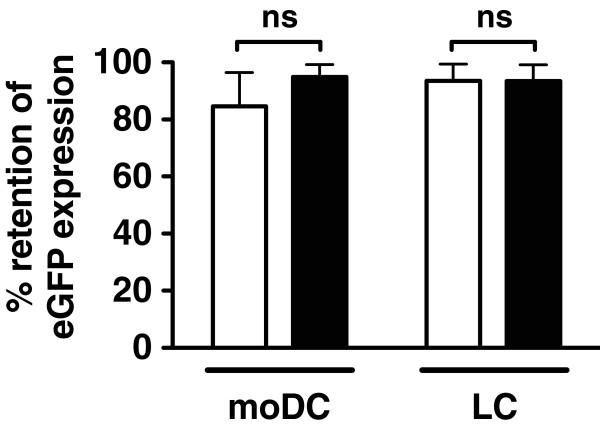
**mRNA-electroporated moDCs and LCs retain eGFP expression after cryopreservation.** Immature (□) and partially-matured (▤) moDCs and LCs were electroporated with eGFP mRNA and cryopreserved. After thawing, eGFP expression for each experimental group was measured by flow cytometry and compared with pre-cryopreservation eGFP expression values to determine percent retention of eGFP expression. Pooled data are shown for each experimental group (mean ± SD, n = 3 independent experiments, ns = not significant).

Immature and mature moDCs and LCs were electroporated with eGFP mRNA, and post-electroporation eGFP expression was determined by flow cytometry. Cells were then cryopreserved, and post-thaw eGFP expression was compared with expression prior to cryopreservation. Retention of protein expression was similar between immature and mature moDCs and LCs (Figure 5).

### mRNA-electroporated moDCs and LCs remain potent stimulators of allogeneic T cells in mixed leukocyte reactions

**Figure 6:**
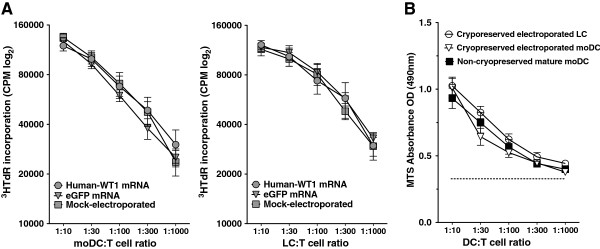
**mRNA-electroporated moDCs and LCs remain potent inducers of allogeneic T cell proliferation, including after cryopreservation and thawing. (A)** MoDCs and LCs, as indicated on the x axes, were electroporated with WT1 mRNA (○), eGFP mRNA (△), or mock-electroporated with no mRNA (□). After electroporation, DCs were terminally matured for 24 hours and then cultured with allogeneic T cells for five days in allogeneic MLRs. DC:T ratios ranged from 1:10 to 1:1000. [3H]TdR uptake by proliferating allogeneic T cells over the final 8 hours of culture was measured as an index of DC immunogenicity (triplicate means ± SEM, n = 3 independent experiments). **(B)** To assess preservation of allo-stimulatory capacity in MLRs after cryopreservation and thawing, WT1 mRNA-electroporated moDCs (△) and LCs (○) were compared with non-cryopreserved mature moDCs (▤). Culture conditions were otherwise exactly the same as in **(A)**. T cell proliferation was measured by a colorimetric assay (triplicate means ± SEM, n = 3 independent experiments). Dotted line represents T cells alone.

Having shown that mRNA-electroporated moDCs and LCs possess the ability to respond normally to inflammatory cytokines by upregulating phenotypic markers of maturation and activation, we further assessed their functional capacity to stimulate resting allogeneic T cell proliferation. While distinct DC subtypes have different capacities to stimulate CD8^+^ CTLs [14,15], their comparable stimulation of bulk allogeneic T cell proliferation in MLRs remains a standard assay of overall DC immunogenicity [14]. MoDCs and LCs were therefore electroporated with eGFP or WT1 mRNA, or were mock electroporated with no mRNA and then matured with inflammatory cytokines for 24 hours. The resulting mature moDCs (Figure 6A, left panel) or LCs (Figure 6A, right panel) were added in serial 3-fold dilutions to a fixed number of purified allogeneic T cells and incubated for 5 days. Allogeneic T cell proliferative responses were measured by [3H]TdR uptake over the last 8 hours of a 5-day culture. Responder T cells showed similar trends of proliferation when cultured with moDC and LCs electroporated under the three experimental conditions (Figure 6A). DCs also retained their allo-stimulatory capacity after cryopreservation and thawing, as measured by a colorimetric proliferation assay (Figure 6B). Thus, both moDCs and LCs remain functionally potent stimulators of allogeneic T cell proliferation, unaffected by prior mRNA electroporation or cryopreservation and despite their differences in CTL stimulation.

## Discussion

This study establishes different conditions for the successful electroporation and expression of full-length mRNA in human CD34^+^ HPC-derived LCs versus blood moDCs, for use in clinical DC-based vaccine trials. Electroporation efficiency depends on the maturation status of the two DC subtypes, with partially-matured LCs showing more efficient transfection than immature LCs and immature moDCs showing more efficient transfection than mature moDCs. Electroporation favorably induces the maturation of CD83^+^HLA-DR^bright^ LCs, whereas electroporation has no direct effect on moDC maturation. LCs and moDCs are equally sensitive to the direct effects of electroporation with decreased viability immediately after electroporation. LCs and moDCs also demonstrate similar retention of transgene expression after cryopreservation. Importantly, both DC subtypes maintain potent immunogenicity after electroporation for stimulating allogeneic T cell proliferation in MLRs, despite their inherent differences in capacity for CD8^+^ CTL stimulation [14-17].

For moDCs, our observation of greater transfection efficiency of immature cells than for mature cells differs from another study in which the transfection efficiency of immature and mature moDCs was roughly equivalent [18]. Various electroporation conditions with disparate degrees of transfection efficiency, however, have been reported for moDCs [8,19-21]. These discrepancies underscore the importance of revalidating electroporation parameters for any differences in electroporation machine, voltage, number of pulses, cell concentration, or amount of mRNA, as depicted in Figure 2.

Peak transfection efficiency is lower for LCs than for moDCs. Likely contributing to this difference is that moDCs are derived from a more differentiated and committed starting population than are LCs, resulting in greater cell population purity. Thus the difference in absolute transfection values would have favored moDCs, as the LCs would have had other contaminating, non-LCs competing for mRNA uptake during electroporation. Purification of LCs by HLA-DR selection on magnetic beads is one potential solution to increase transfection efficiency, but this should not be necessary to the extent that one can quantify the proportion of electroporated LCs by flow cytometry in the final antigen-presenting cell population.

Consistent with previous studies [22-24], the immediate post-electroporation viability of mRNA-electroporated DCs is less than the initial starting population. Subsequent survival of viable cells is minimally affected, however. Cryopreservation can further decrease cell recovery, so an appropriate excess of electroporated DCs should be aliquoted for cryopreservation. This will ensure sufficient DC yields, irrespective of moDC or LC subtype, from thawed aliquots for vaccination.

We previously reported the results of a clinical trial in AJCC stage III/IV melanoma patients, comparing vaccination with peptide-loaded LCs versus peptide-loaded moDCs [12]. The rationale for this trial was based on our established findings *in vitro* that human LCs are consistently superior to moDCs on a cell-for-cell basis in eliciting MHC-restricted, antigen-specific CTLs [14,16], which has been corroborated by other investigators [15,17]. A clinical trial conducted by Banchereau and colleagues for similarly advanced-stage melanoma patients using bulk CD34^+^ HPC-derived DCs, which included LCs, pulsed with a mixture of melanoma-derived peptides, resulted in durable immune responses associated with long-term survival [25]. Although patients treated on the LC arm of our trial generated significantly greater reactivity against tyrosinase than those treated with moDCs, significant differences were not observed for gp100 or the control fluMP antigen [12]. This begged the obvious question as to whether loading class I MHC-restricted single peptides onto any defined DC subtype would ever be sufficient to stimulate durable immunity against tumor antigens.

Our group has since discovered that LCs from healthy volunteers, electroporated with Wilms’ tumor 1 (WT1) mRNA, promote sustained presentation of antigenic peptides, which in conjunction with IL15R-α/IL15, induce robust autologous, WT1-specific CTLs [16]. The CTLs develop after just 7 days’ stimulation without exogenous cytokine supplementation and lyse MHC-restricted targets, including primary WT1-expressing blasts from leukemia patients. MoDCs, in contrast, require exogenous IL15 to promote immune responses comparable to LCs. Thus LCs provide a more favorable cytokine milieu for the activation of T cells than do moDCs, thereby explaining their superior induction of antigen-specific CTLs in the absence of exogenous cytokines. These data support the use of mRNA-electroporated LCs, or IL15-supplemented moDCs, as cancer vaccines to overcome tolerance against self-differentiation tumor antigens. The pivotal role of IL15 in LC-mediated stimulation of CTLs has also been confirmed using individual peptides rather than mRNA encoding full-length protein [17,25].

Given the importance of LCs and their provision of IL15, as well as the possible application of IL15-supplemented moDCs in DC-based vaccines, we thought it essential to ascertain the optimal conditions for their respective electroporation with mRNA to express full-length protein. Such expression enables cells to process and present antigenic peptides tailored to their own MHCs, allowing clinical investigators to move beyond single defined peptides for a given tumor, if they are even known, and circumvent the limitations of specific HLA types in patients. There may be additional, as yet undiscovered reasons for LCs’ potency over moDCs, which may nonetheless prove sufficiently compelling to favor LCs in vaccine trials. MoDCs offer the potential benefit of activating NK cells via IL12p70 [26]. LCs do not secrete IL12p70 but maintain NK cell viability via IL15 [14,26]. It will be important to determine whether moDCs supplemented with IL15 are an adequate substitute for LCs, or whether the two DC subtypes should be used in combination to capitalize on the efficacy of moDCs in activating NK cells and the potency of LCs in inducing CD8^+^ CTLs.

## Conclusions

Enhanced antigen presentation by DCs can be achieved with mRNA electroporation but requires modifying conditions to the specific DC subtype. Our findings with LCs and moDCs provide key parameters on the optimal timing of mRNA electroporation, effects on cell maturation, and anticipated cell losses and yields. Investigators should now have the tools in hand to address key questions about DC-based cancer immunotherapy *in vivo* in humans, with reasonable maintenance of LC or moDC viability and preservation of the activated phenotype and baseline immunostimulatory capacity.

## Abbreviations

APC: Antigen-presenting cell; CTL: Cytotoxic T lymphocyte; DC: Dendritic cell; eGFP: Enhanced green fluorescent protein; FLT-3-ligand: Fms-related tyrosine kinase-3-ligand; fluMP: Flu matrix peptide; G-CSF: Granuloctye colony-stimulating factor; GM-CSF: Granuloctye macrophage colony-stimulating factor; HPC: Hematopoietic progenitor cell; IL-1β: Interleukin-1-beta; IL-6: Interleukin-6; IL12p70: Interleukin-12p70; IL15: Interleukin-15; IL15R-alpha: Interleukin-15-receptor-alpha; LC: Langerhans-type DC; MHC: Major histocompatibility complex; MLR: Mixed leukocyte reaction; moDC: Monocyte-derived DC; NHS: Normal human serum; NK cell: Natural killer cell; pSTAT5: Phosphorylated signal transducer and activator of transcription 5; TGF-β: Transforming growth factor-beta; TNF-α: Tumor necrosis factor-alpha; WT1: Wilms’ tumor 1

## Competing interests

The authors have no competing interests to declare.

## Authors’ contributions

DJC designed and performed experiments, analyzed and interpreted data, and wrote the manuscript. ER designed and performed experiments, analyzed and interpreted data, and made comments on the manuscript. ER and MM designed the WT1-encoding plasmid. KBP, JAS, and ETSA performed experiments. JWY designed the overall study, supervised performance of experiments, analyzed and interpreted data, and wrote the manuscript. All authors read and approved the final manuscript.
